# Deep Learning Based Switching Filter for Impulsive Noise Removal in Color Images

**DOI:** 10.3390/s20102782

**Published:** 2020-05-14

**Authors:** Krystian Radlak, Lukasz Malinski, Bogdan Smolka

**Affiliations:** 1 Faculty of Automatic Control, Electronics and Computer Science, Silesian University of Technology, 44100 Gliwice, Poland; bogdan.smolka@polsl.pl; 2 Faculty of Materials Engineering and Metallurgy, Silesian University of Technology, 40019 Katowice, Poland; lukasz.malinski@polsl.pl

**Keywords:** deep learning, deep neural networks, image denoising, image enhancement, impulsive noise, switching filter

## Abstract

Noise reduction is one of the most important and still active research topics in low-level image processing due to its high impact on object detection and scene understanding for computer vision systems. Recently, we observed a substantially increased interest in the application of deep learning algorithms. Many computer vision systems use them, due to their impressive capability of feature extraction and classification. While these methods have also been successfully applied in image denoising, significantly improving its performance, most of the proposed approaches were designed for Gaussian noise suppression. In this paper, we present a switching filtering technique intended for impulsive noise removal using deep learning. In the proposed method, the distorted pixels are detected using a deep neural network architecture and restored with the fast adaptive mean filter. The performed experiments show that the proposed approach is superior to the state-of-the-art filters designed for impulsive noise removal in color digital images.

## 1. Introduction

Image denoising is a long-standing research topic in low-level image processing that still receives much attention from the computer vision community [1,2]. Over the last three decades, a considerable increase in the effectiveness of algorithms took place, but despite these improvements, modern miniaturized high-resolution, low-cost image sensors still provide a limited quality, when operating in poor lighting conditions. Therefore, image enhancement and noise removal are very important operations of digital image processing [3,4].

In practice, we can observe various types of noise that significantly degrade the quality of captured images. One of them is the so-called *impulsive noise*, which may appear due to electric signal instabilities, corruptions in physical memory storage, random or systematic errors in data transmission, electromagnetic interferences, malfunctioning or aging of camera sensors and low lighting conditions [3,5,6,7]. This type of noise causes a total loss of information at certain image locations because the original color channels information is replaced by random values.

In the literature, impulse noise is typically classified into two main categories [8,9,10]. The first one is the Channel Together Random Impulse (CTRI), in which a pixel channel may be replaced with any value in the image intensity range. The second noise model is salt and pepper impulse noise, in which a corrupted pixel value is set to either the minimum or the maximum of a range of possible values (so it is set to either 0 or 255 for an 8-bit image). In both models, the main parameter is the noise density ρ, which is the fraction of corrupted pixels in the processed image. In this paper, we focus on the CTRI model, however the proposed filter can also be applied to the salt and pepper model.

The classical method for removal of impulsive noise is the median filter. Generally, the median concept for color images is based on vector ordering, in which image pixels are treated as three-dimensional vectors. Such an approach yields better results than processing image channels independently [11,12,13,14], as the strong correlation between color channels is considered. The most widely used denoising method based on the ordering concept is the Vector Median Filter (VMF) [15], whose drawback is that every pixel of the image is processed, regardless of whether it is contaminated or not. This may result in strong signal degradation and introduction of perceivable blurring effect, especially in highly textured regions. In many applications, it may become a major flaw, and therefore, a plethora of improvements have been proposed [6,16,17,18,19,20,21,22,23,24,25].

**Figure 1:**
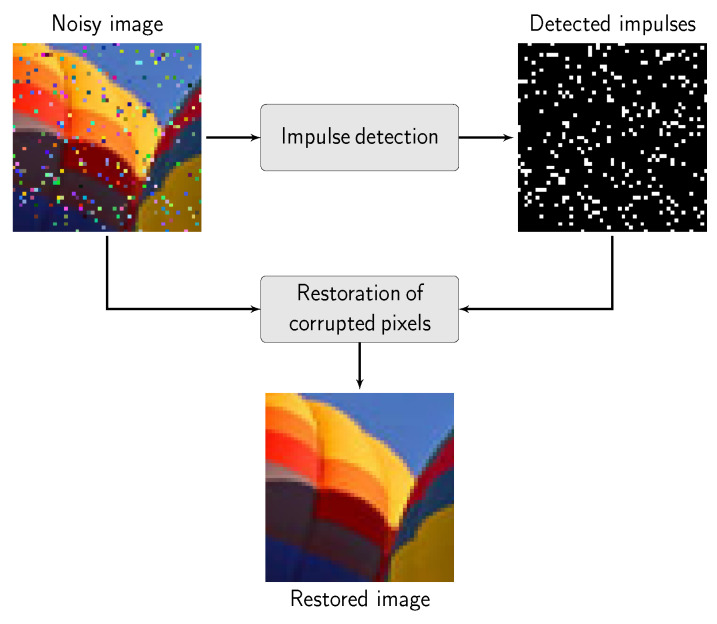
A general scheme of a switching filter. Only the pixels identified as corrupted are being restored and the remaining pixels are retained.

To preserve image details and still efficiently suppress impulsive noise, a family of filters based on fuzzy set theory was also introduced, in which a combination of noise detection and a replacement scheme based on weighted averaging is performed [26,27,28,29,30,31]. However, these methods still may alter clean pixels in the processed image. An effective approach to retain uncorrupted pixels is based on the switching concept [32]. A general scheme of switching filter is presented in Figure 1. In the majority of the switching techniques, it is necessary to determine the measure of the impulsiveness of the processed pixel, which allows classifying the pixels as pristine or distorted. One of the most popular measures of similarity used in switching filters is the ROAD (Rank-Ordered Absolute Differences) statistic introduced in [33], in which the trimmed cumulative distance of the pixels, in a given color space, to their neighbors is utilized as a degree of pixel corruption.

Among switching filters, an important group of methods is based on the concept of a peer group [34,35,36], in which the membership degree of a central pixel of the filtering window to its local neighborhood is determined in terms of the number of close pixels. Another efficient family utilizes the elements of quaternion theory [37,38]. In this concept, instead of the commonly used Euclidean distance in a chosen color space, the similarity between pixels is defined in the quaternion form. Some switching filters also use classical machine learning approaches for impulse detection such as Support Vector Machines [39] and fully connected neural networks [40,41,42,43]. The detected impulses are restored using a median of neighboring uncorrupted pixels [40,42], adaptive and iterative mean filters [41] or edge-preserving regularization methods [43].

Recently, thanks to easy access to large image datasets and advances in deep learning, the Convolutional Neural Networks (CNNs) have led to a series of breakthroughs in various computer vision problems such as image segmentation, object recognition and detection. Concurrently, CNNs have also been successfully applied for image denoising, focusing mainly on the problem of Gaussian noise suppression. The recently proposed filters significantly outperform classical, well established algorithms in terms of filtering efficiency and computational speed [44,45,46]. However, despite the fact that image denoising using deep learning for Gaussian noise removal has been well-studied, relatively little work has been done in the area of impulsive noise detection and removal [47,48,49,50,51]. In [47], the authors proposed a method, which replaces noisy image pixels by a weighted average of samples from the neighborhood to remove salt and pepper noise and then the filter output is further processed using CNN to boost the final filtering performance. An alternative technique [48] divides the input image into small patches, which are processed independently by a set of convolution and deconvolution layers. Yet another approach [50] is based on a CNN trained on noisy images and is using the ROAD statistic to estimate the contamination level and to select the network found to be optimal for the estimated image pollution. The authors of [51] proposed to use two CNNs: the first one detects noisy pixels and the second one performs the final image reconstruction.

In one of the most promising approaches, called Denoising Convolutional Neural Network (DnCNN) [52], the authors proved that residual learning and batch normalization are beneficial in the case of the Gaussian noise model. Unfortunately, the network based on residual learning formulation is not effective in the case of other types of operations like JPEG artifacts removal, deblurring or image resolution enhancement [53] and also in the case of impulsive noise suppression. Applying residual learning for images contaminated by impulsive noise causes all pixels in the image to be altered, even those that were impulse-free, introducing unpleasant visual artifacts. Moreover, impulsive noise is not additive, and therefore, a residual approach is not suitable. To alleviate the inability of DnCNN to cope with impulsive distortions, we propose a modified version, which will be denoted as Impulse Detection Convolutional Neural Network (IDCNN).

In the proposed approach, we added a sigmoid layer to distinguish noise-free pixels from impulses and we reformulated the residual learning to the classification problem. In this way, the deep neural network is used as the impulse detector, and afterward, the corrupted pixels are restored using an adaptive mean filter, due to its good balance between computational complexity and restoration efficacy. The main contributions of our paper are as follows:

- We introduce a CNN architecture for impulse detection in noisy images,
- We propose a switching filter that applies deep learning for the localization of corrupted pixels and adaptive mean technique for their replacement,
- We analyze the impact of some network’s parameters on impulse detection accuracy,
- We also investigate the influence of impulse detection errors on the final restoration efficiency,
- We show that the proposed method is superior to the state-of-the-art denoising algorithms,
- We share the source code of the proposed approach at http://github.com/k-radlak/IDCNN.

The paper is organized as follows. Section 2 describes the structure of the proposed switching filter, focusing on the architecture of the proposed IDCNN and also presents its ablation study and the applied noisy pixel replacement method. The next section presents a comparison of the proposed technique with state-of-the-art filters designed for impulsive noise removal. Finally, discussion and conclusions are given in Section 4.

## 2. Proposed Switching Filter Design

Recently, the application of deep learning for image denoising has received much attention from the computer vision community due to its significant performance improvement in comparison to the classical machine learning algorithms. One of the most interesting methods, intended for Gaussian noise suppression, is the Denoising Convolutional Neural Network (DnCNN), inspired by VGG network [54] and introduced by Zhang et al. [52].

The DnCNN contains a sequence of convolutional layers followed by Rectified Linear Unit (ReLU) [55] and Batch Normalization (BN) [56]. The first layer has a convolution filter and ReLU activation. The second and each consecutive layer consists of a convolution filter, BN and ReLU activation, with the exception of the last layer that only uses convolution. The training of the network is based on the concept of deep residual learning [57], in which the network does not estimate the original values of the undistorted image, but instead learns to estimate the difference between a noisy and clean image.

The DnCNN filter outperforms most of the state-of-the-art algorithms designed for Gaussian noise reduction, but due to the fact that it uses residual learning, the original DnCNN trained on impulsive noise model also alters non-corrupted pixels as was shown in [49]. Therefore, in the proposed IDCNN, we modified the original DnCNN architecture to ensure that noise-free pixels will not be affected.

### 2.1. Impulsive Noise Detection Using CNN

**Figure 2:**
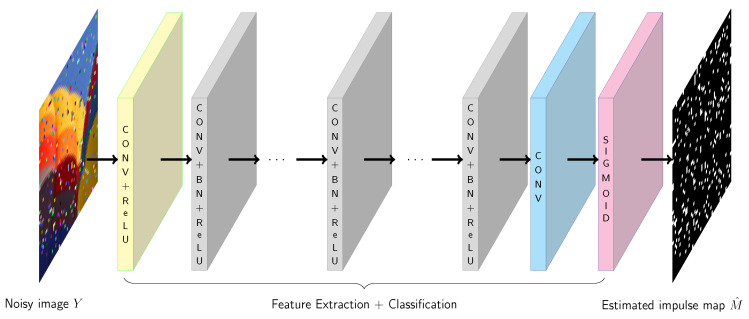
The architecture of the proposed network for impulsive noise detection.

In our approach, instead of the usage of residual learning, we employed all layers proposed in DnCNN for feature extraction and we added a sigmoid layer, which estimates for each pixel its impulsivity measure π∈[0,1], the values of which are close to 0 when a pixel is likely to be undistorted and close to 1 if it seems to be corrupted. In this way, the network divides the image pixels into clean and distorted, depending on the appropriate value of the π measure. The pixels with π=0 or π=1, are treated by the network as clean or corrupted respectively, with highest confidence. The architecture of the proposed network is depicted in Figure 2.

**Figure 3:**
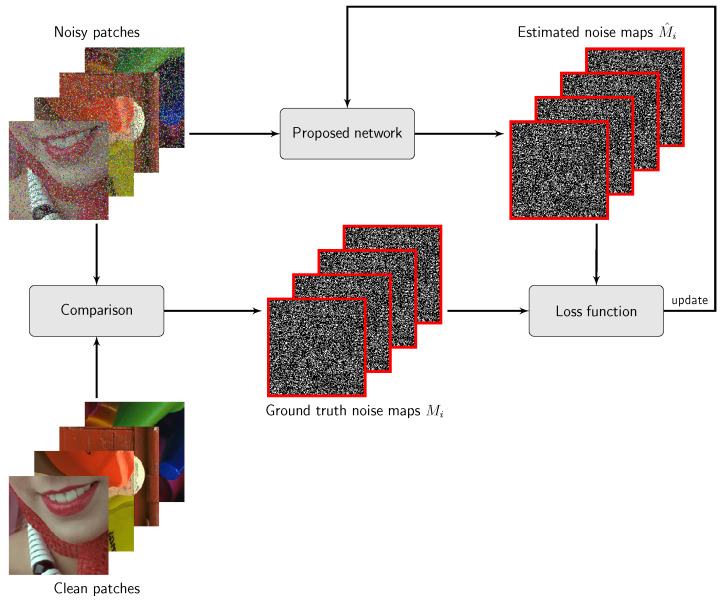
Training of the proposed Impulse Detection Convolutional Neural Network (IDCNN) detector.example

The introduced architecture also requires a change in the training procedure. In the original DnCNN, during the training, the images are divided into small, square and non-overlapping patches of size p×p. The loss function is then determined taking into account the clean and denoised patches. In our approach, we generate a ground truth noise map *M* (consisting of values: 0 for clean pixels and 1 for impulses). Then, in the training procedure we calculate the loss function utilizing the patches cropped from *M* and the noise map $\hat{M}$, whose intensities are in the range [0,1], estimated by the network, as shown in Figure 3. More formally, the loss function is defined as

(1) $$L\left(\Theta \right)=\frac{1}{N}\sum_{i=1}^{N}{\left(M_{i}-\hat{M_{i}}\right)}^{2},$$

where Θ denotes a set of trainable parameters, $M_{i}$, $\hat{M_{i}}$ denote for each patch *i* the original and the estimated noise map, respectively, and *N* stands for the number of patches used in the training. Finally, the output of the IDCNN is a map of impulsivity measure π that has to be binarized using a threshold, to finally classify a pixel as either noisy or undistorted.

### 2.2. Detected Noisy Pixels Replacement

In order to restore the detected noisy pixels, we used the modified version of the adaptive arithmetic mean filter introduced in [41], which offers satisfying image quality and reasonable computational speed. This algorithm of restoration of the detected noisy pixel can be summarized as follows:

1. Select initial window of size W=3×3 centered at the detected noisy pixel and calculate the number of uncorrupted pixels. If all pixels are corrupted, then go to step 2, otherwise go to step 4.
2. Increase the size of *W* by 2.
3. Calculate the number of uncorrupted pixels in *W* and if all pixels are corrupted, then go to step 2, otherwise go to step 4.
4. Replace the processed pixel by the average of clean pixels inside *W*.

The proposed algorithm restores only pixels that were classified by the network as impulses. However, it is worth mentioning here that the corrupted pixels can be replaced using other, more efficient techniques, for example, an image restoration algorithm based on deep neural network introduced in [58]. This issue will be the subject of follow-up research.

### 2.3. Network Training and Ablation Study

In order to evaluate the performance of the proposed network, we started with the default parameters that were proposed for DnCNN [52]. These parameters are summarized in Table 1. Additionally, for training purposes, all images were resized using bicubic interpolation in four scales {1,0.9,0.8,0.7} and we also performed data augmentation: image rotations (90°, 180°, 270°) and flipping in the vertical direction. Here, it is worth mentioning that the small patches were used only in the training phase, but in inference, the obtained convolution masks were applied to the whole image.

**Figure 4:**
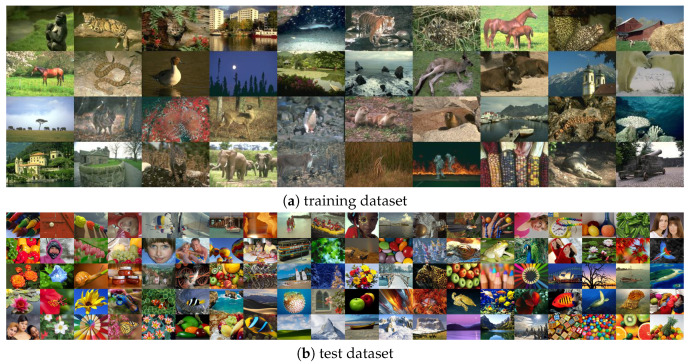
Example images from (**a**) Berkeley segmentation dataset (BSD500) [61] used for training purposes and (**b**) our test dataset accessible from [36] as supplementary material.

In our experiments, we used a Berkeley segmentation dataset (BSD500) [61] that consists of 500 natural images in resolution 481×321. Exemplary images from BSD500 are depicted in Figure 4a.

For testing purposes, we used the dataset introduced in [36], consisting of 100 color images in resolution 640×480, which are presented in Figure 4b. In this paper, all presented results were obtained on this dataset.

For training purposes, these images were contaminated using the CTRI model. In this model each RGB pixel $x_{i}=\left(x_{i}^{1},x_{i}^{2},x_{i}^{3}\right),i=1,2,\ldots ,Q$, is contaminated with probability ρ and each channel of the noisy pixel $y_{i}$ obtains new values from the range [0,255] drawn from a uniform distribution. Index *i* determines the pixel position on the image domain and *Q* is the total number of image pixels. This model can be formally defined as

(2) $$y_{i}=\left\{\begin{array}{cc} \xi_{i}, & \mathrm{with}\mathrm{probability}\rho ,\\ x_{i}, & \mathrm{with}\mathrm{probability}1-\rho , \end{array}\right.$$

where $\xi_{i}=\left(\xi_{i}^{1},\xi_{i}^{2},\xi_{i}^{3}\right)$ denotes the contaminated pixel, $\xi_{i}^{k}\in \left[0,255\right],k\in \left\{R,G,B\right\}$.

In order to evaluate the noise detection efficiency of the proposed network and impact of the network’s parameters, we propose to transform the problem into the classification domain, instead of using traditional measures for image denoising. In the proposed evaluation methodology, the result of noise detection is represented by the estimated noise map $\hat{M}$ and is compared to the ground truth map *M*. Then, impulsive noise detection problem can be transformed into noisy vs. clean pixels classification and the results can be presented using the number of True Positives (TP), True Negatives (TN), False Positives (FP), False Negatives (FN):

- TP are pixels that were correctly recognized as impulses,
- TN are pixels that were correctly recognized as clean,
- FP are pixels that were incorrectly classified as noisy,
- FN are pixels that were incorrectly classified as uncorrupted.

Finally, the network performance can be evaluated using weighted accuracy (wACC) defined as

(3) $$\mathrm{wACC}=\left(1-\rho \right)\frac{\mathrm{TP}}{\mathrm{TP}+\mathrm{FN}}+\rho \frac{\mathrm{TN}}{\mathrm{TN}+\mathrm{FP}}.$$

Weighted accuracy considers the number of pixels that were correctly classified when the classes are unbalanced and their cardinalities depend on selected noise intensity ρ. However, this metric does not distinguish the type of errors made by the network, if an impulse was missed or incorrectly classified as pristine. To better analyze the detection performance, we evaluated the False Positive Rate (FPR) defined as

(4) $$\mathrm{FPR}=\frac{\mathrm{FP}}{\mathrm{FP}+\mathrm{TN}},$$

which shows the ratio of incorrectly classified clean pixels as impulses to the total number of clean pixels in the processed image. Additionally, we made use of the False Negative Rate (FNR) defined as

(5) $$\mathrm{FNR}=\frac{\mathrm{FN}}{\mathrm{TP}+\mathrm{FN}},$$

which represents the ratio of wrongly detected noisy pixels to the total number of impulses. Another measure used to evaluate detection performance is the F_1_-score, defined as

(6) $$F_{1}=2\frac{\mathrm{precision}\times \mathrm{recall}}{\mathrm{precision}+\mathrm{recall}},$$

where precision = TP/(TP+FP) and recall = TP/(TP+FN). This measure does not take into account the cardinality of TN results, because the number of TN tends to be dominant in most cases of impulsive noise detection. Therefore, it is more sensitive to detection imperfections than wACC.

**Figure 5:**
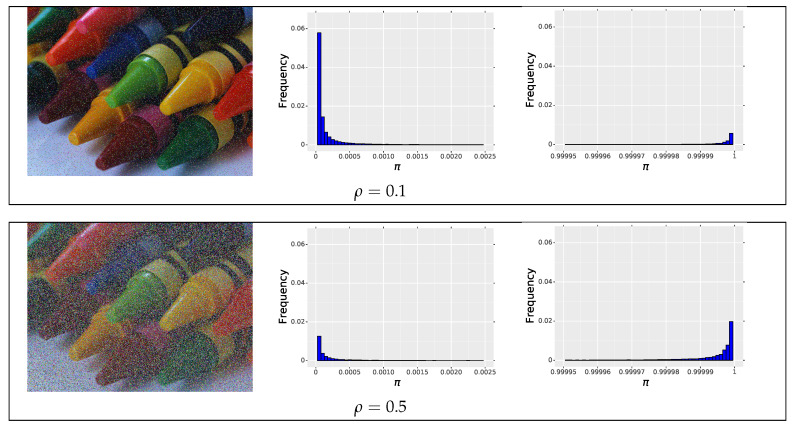
Exemplary distributions of the IDCNN outputs for the test image contaminated with impulsive noise with intensity ρ=0.1 and 0.5. For the contamination ρ=0.1, 89.59% of pixels are assigned π<0.1 and 10.14% reaches a value π>0.9. For noise intensity ρ=0.5, the respective frequencies are 50.20% and 49.44%.

To evaluate the network performance on the whole test dataset, we determined wACC, TPR, FPR and F_1_-score for each image and then calculated their average values. To correctly localize impulses in the image using the output of the proposed IDCNN, in the first step it is necessary to estimate the proper value of the threshold, to decide which pixels are contaminated. Selection of the optimal threshold typically can significantly affect the final results, but we noticed that the values of the impulsivity degree π returned by the network are very close to 0 if a pixel is clean and close to 1 if a pixel is likely to be an impulse. Exemplary distributions of π measures returned by the IDCNN are presented in Figure 5, where we show only the left and right part of the histogram. Therefore, in our research, we set the threshold, which divides the pixels into clean and noisy to 0.5 as the values of impulsiveness are very close to 0 or 1.

**Figure 6:**
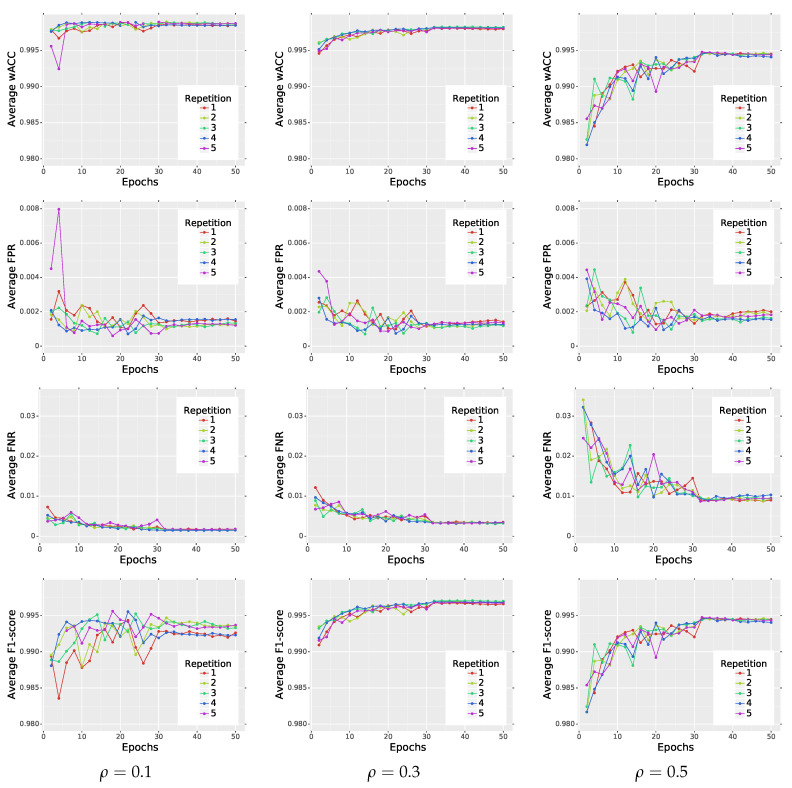
Repeatability of the training procedure on BSD500 dataset.

In order to better understand the influence of different parameters on the final performance of the proposed network, we conducted some additional experiments. In the first one, we checked whether the network impulse detection efficiency is repeatable when we start the training procedure from scratch. The changes of the average wACC, FPR, FNR and F_1_ score calculated on test database during the training are presented in Figure 6 and in Table 2. The outcomes are repeatable and the network starts to stabilize and converges to its final performance when the learning rate is decreased after 30 epochs. Additionally, we can see that the average FPR is relatively low and wACC reflects the network’s performance quite well. Therefore, in the rest of the paper, we present the results of the wACC metric only.

**Figure 7:**
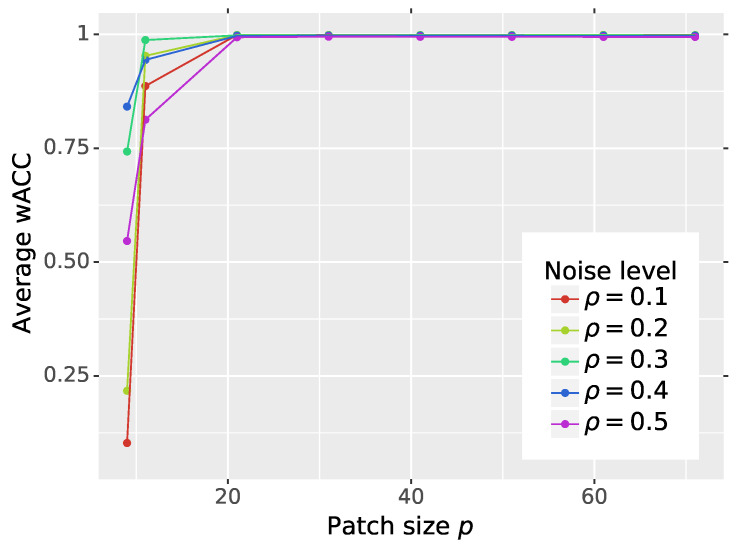
Impact of the patch size *p* used in the training procedure on the network’s weighted detection accuracy.

In the next experiment, we evaluated the influence of the patch size *p* used in the training procedure on the final average performance (see Figure 7 and Table 3). We tested our method using the following patch sizes *p*: {9,11,21,31,41,51,61,71}. For smaller *p*, the network was not able to learn, and therefore, the results are not presented.

As can be observed, if the patch size used in the training is not smaller than 21×21, the optimal performance of the network is achieved. However, the increase of the patch size does not boost the network’s performance, but it makes training more time consuming, because the loss function is calculated for bigger patches. Therefore, the selected patch size cannot be too small nor too big as it would only increase the training time.

**Figure 8:**
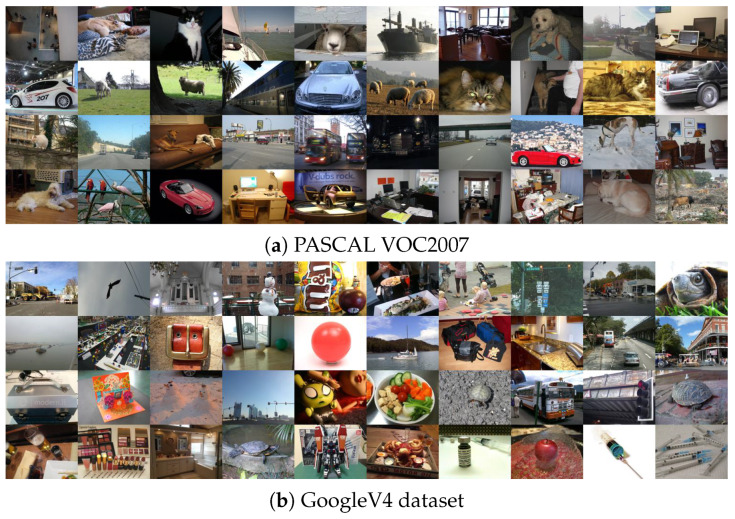
Example images from GoogleV4 and PASCAL VOC2007 datasets [63].

In the next experiment, we analyzed the impact of the type of dataset used in training procedure and its size on the final network performance. We selected two additional datasets: the PASCAL VOC2007 dataset [62] and the Google Open Images Dataset V4 (GoogleV4) [63] used for object detection purposes. Both datasets contain high quality images and we selected randomly 500 pictures. The PASCAL VOC2007 dataset consists of images, which present 20 classes of various objects. The GoogleV4 dataset contains images, which cover 600 classes. From the GoogleV4 dataset we randomly selected 50 classes and additionally we also decreased the original resolution four times to ensure similar image sizes in all training and test datasets. Example images from both datasets are presented in Figure 8.

**Figure 9:**
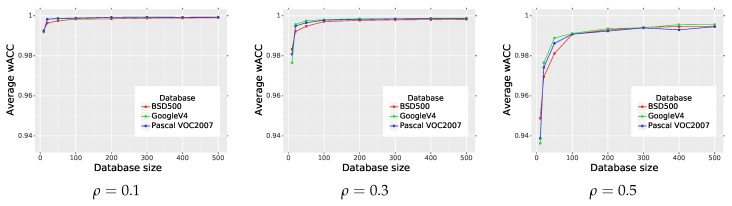
Impact of the type of dataset used in the training and its size on the average wACC of the proposed IDCNN.

The influence of the size of the training dataset on the final average performance of the network is shown in Figure 9 and summarized in Table 4. As can be observed, if the size of the dataset is increased, then the average wACC is also growing. The highest average wACC was achieved for GoogleV4 dataset, but the difference between various datasets is rather small. The performed experiment also shows that the training of the network requires sufficient amount of data to reach expected effectiveness. However, the optimal performance can be achieved on different datasets. Additionally, we can notice that when the noise density increases, the network needs more data in the training.

Finally, we recommend to use 500 images in the training, but we need to remember that the final number of patches can differ depending on image resolution. In a single experiment, the total number of non-overlapping patches of size 41×41 generated for BSD500 dataset was 120,500.

**Figure 10:**
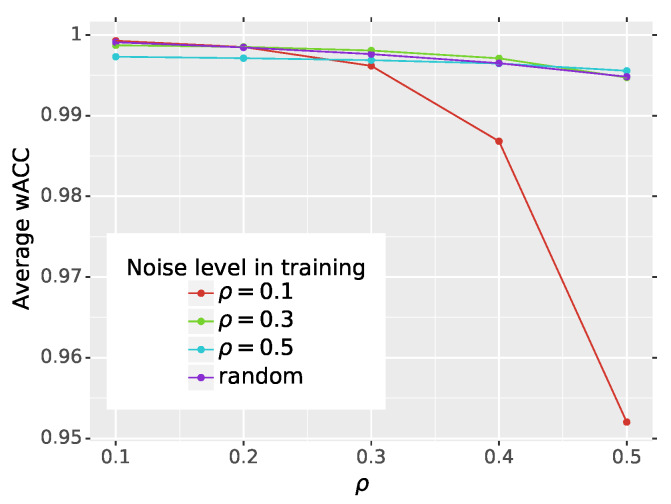
Impact of the noise density used during training on the final network performance.

The last issue that we would like to address in the scope of network learning is the noise level that should be used in the training procedure to obtain optimal network performance. For the original DnCNN, the authors proved that the efficiency of their network was not influenced by the Gaussian noise intensity. To evaluate this behavior for impulsive noise, we trained the network with patches contaminated with noise density ρ={0.1,0.3,0.5}. Additionally, we trained the network with patches contaminated with randomly selected noise probability from the range [0.1,0.5]. This experiment is denoted in this paper as “random” and the results are depicted in Figure 10 and summarized in Table 5.

It can be noticed that the highest values of wACC are obtained if the noise level during training and tests is the same. When using a random noise density during training, the results are very close to the optimal performance. The highest deviations from the optimal average wACC were obtained for low noise contamination level during training and heavy noise at testing phase and vice versa. In most cases, the maximum of wACC was achieved using ρ=0.3 and for other noise levels in the test phase, the performance was very close to the optimal one. Therefore, we recommend to use this value during training. In the future, it will be interesting to investigate the impact of the diversity of the dataset used during training on the final network performance.

## 3. Comparison with the State-of-the-Art Denoising Methods

The proposed switching filter was compared with the competitive methods in two variants of IDCNN trained on different datasets: BSD500 (IDCNN_B_) and GoogleV4 (IDCNN_G_). The state-of-the-art algorithms chosen for comparison are listed bellow:

- Adaptive Weighted Quaternion Color Distance (AWQD) [64],
- DnCNN trained on impulsive noise model [49],
- Fast Averaging Peer Group Filter (FAPGF) [36],
- Fast Adaptive Switching Trimmed Arithmetic Mean Filter (FASTAMF) [32],
- Fast Fuzzy Noise Reduction Filter (FFNRF) [29],
- Fuzzy Rank-Ordered Differences Filter (FRF) [65],
- Fuzzy Weighted Non-Local Means (FWNLM) [66],
- Impulse Noise Reduction Filter (INRF) [67],
- TV-based restoration method with $\ell_{0}$TV-norm data fidelity (L0TV) [68],
- Patch-based Approach for the Restoration of Images affected by Gaussian and Impulse noise (PARIGI) [69],
- Peer Group Filter (PGF) [35],
- Quaternion-Based Switching Filter (QBSF) [70],
- Two-stage Quaternion Switching VMF (TSQSVF) [71],
- Blind Denoising CNN (BDCNN) [72],
- Pixel-shuffle Down-sampling (PD) [73].

The implementation codes were downloaded from the authors’ websites and we used the recommended parameters. In our experiments, we also used DnCNN, trained on the impulsive noise model, because as it was shown in [49], even though DnCNN was designed for Gaussian noise, when it is trained on impulsive noise model, it still might provide competitive results in comparison to the state-of-the-art filters.

### 3.1. Evaluation Using Objective Quality Measures

The assessment of selected filtering techniques performance was done using objective numerical measures. We used Peak Signal to Noise Ratio (PSNR) and the Mean Absolute Error (MAE), defined as:

(7) $$\begin{array}{c} \mathrm{PSNR}=10\log_{10}\left(\frac{255^{2}}{\mathrm{MSE}}\right),\;\;\;\mathrm{MSE}=\frac{1}{3Q}\sum_{i=1}^{Q}\sum_{k\in \{R,G,B\}}\;{\left(x_{i}^{k}-{\hat{x}}_{i}^{k}\right)}^{2},\;\;\;\mathrm{MAE}=\frac{1}{3Q}\sum_{i=1}^{Q}\sum_{k\in \{R,G,B\}}\;\left|x_{i}^{k}-{\hat{x}}_{i}^{k}\right|, \end{array}$$

where $x_{i}^{k}$, ${\hat{x}}_{i}^{k}$ denote the RGB channel values of original and restored pixels. Additionally, we employed the Structural SIMilarity index (SSIM_c_) designed for color images [74], because it has demonstrated better agreement with human perception than traditional metrics.

**Figure 11:**
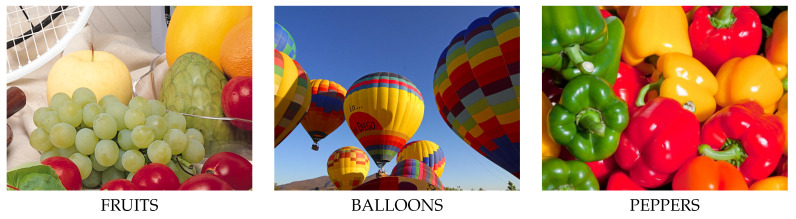
Representative test images from benchmark dataset [36] for which numerical results were calculated.

The numerical results are shown in Table 6 using three representative test images chosen from the dataset [36] presented in Figure 11. To make an analysis of the data in Table 6 and Table 7 more convenient, we annotated five of the best results for each noise level using green color, and bold font was used to indicate the best one. The following remarks can be formulated:

- In all cases, the proposed filter outperforms the state-of-the-art techniques and the IDCNN_G_, trained on GoogleV4 dataset, provided the best results for every image and quality measure.
- The AWQD and FASTAMF filters can be distinguished as those that appear very often among the five best results (depicted in green color).
- Other techniques provide noticeably good results in a rather random manner, thus those may be efficient for certain images, noise ratios or applied quality measure.

**Figure 12:**
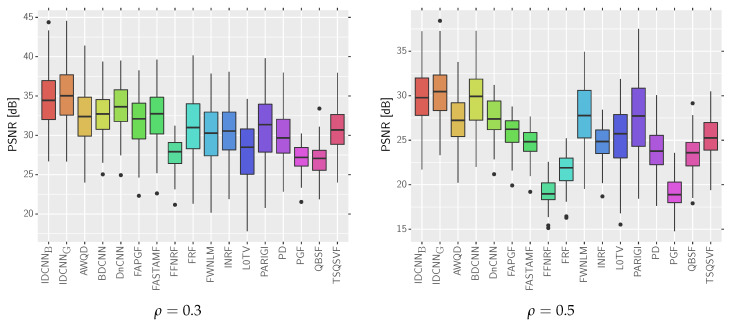
Box plots presenting the distributions of the obtained results for the analyzed methods using the test dataset [36].

Finally, the average values of selected metrics calculated on the test dataset [36] are presented in Table 7. We also included the representative boxplots for PSNR measure to show the distribution of the obtained results (see Figure 12). As can be observed, the average results of all used quality measures are significantly better than state-of-the-arts filters. In addition, the proposed switching filter allows achieving much better results than the original DnCNN trained for impulsive noise.

### 3.2. Visual Assessment

**Figure 13:**
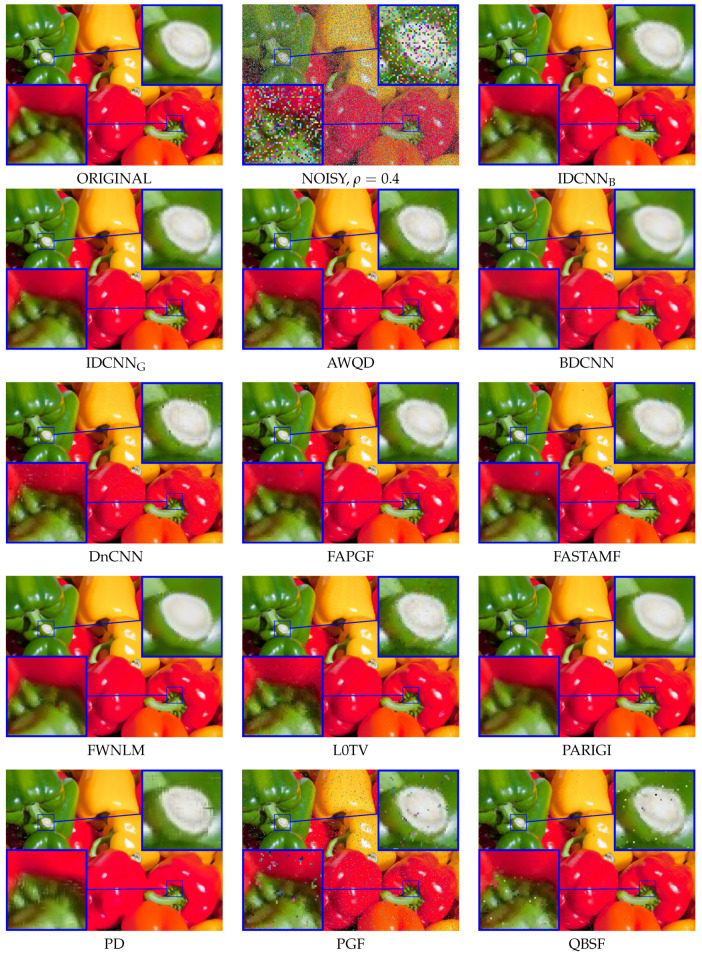
Visual comparison of the filtering efficiency using a part of the PEPPERS image (ρ=0.4).

The visual comparison of the obtained results is depicted in Figure 13. One may notice that the proposed IDCNN is able to correctly localize almost all impulses and the visible artifacts are the effect of insufficient quality of restoration of the noisy pixels. Impressive results for high noise fraction were also obtained applying BDCNN. The main drawback of BDCNN is the fact that it was designed to cope with mixed Gaussian and impulsive noise and therefore uncorrupted pixels in the image were also altered. Due to the smoothing property, this approach sometimes excels over our switching technique for intensive noise in terms of PSNR (Table 6), but the MAE values, which are indicators of detail preservation achieved by our technique, are lower than those obtained with BDCNN. This effect is caused by errors in the detection of noisy pixels made by IDCNN and insufficient quality of the applied noisy pixel replacement method.

**Figure 14:**
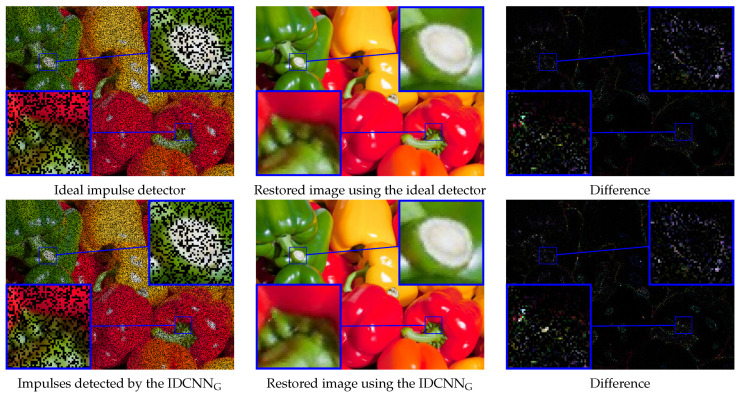
Visualization of the denoising efficiency of the proposed IDCNN_G_ in comparison to the ideal impulse detector, which correctly identifies all impulses in the analyzed image (ρ=0.4). The right column shows the difference between the restored and clean image.

Additionally, in Figure 14, we presented a comparison of the proposed IDCNN with the ideal impulse detector that correctly localizes all impulses in the analyzed image (impulses are localized using ground truth map) and then the corrupted pixels are restored in both cases using the fast adaptive mean filter. As can be observed, only a few impulses (which are very similar to the original texture in the analyzed PEPPERS image) were not correctly detected by the proposed IDCNN network.

**Figure 15:**
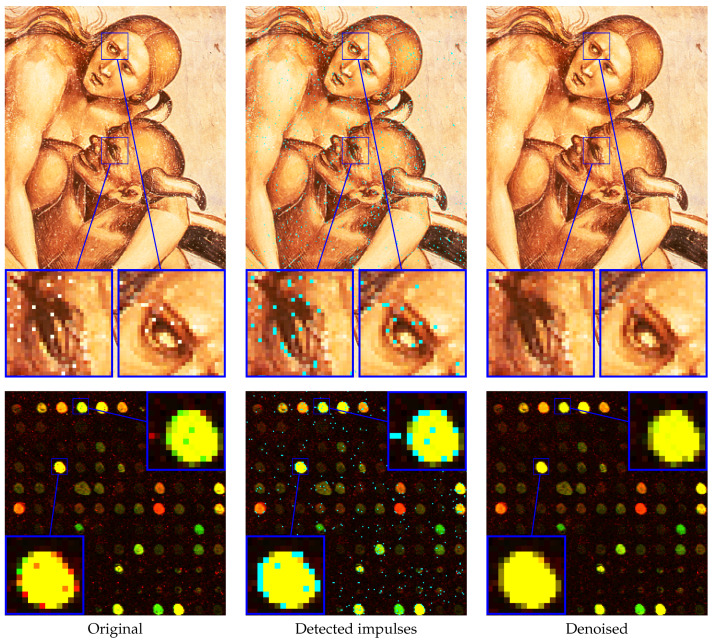
Denoising result of the proposed IDCNN filter on real noisy images: a part of an image of the fresco “The Condemned in Hell” by Luca Signorelli (**top**) and cDNA image (**bottom**). Detected impulses are annotated using cyan color.

The performance of the proposed method was also evaluated on real noisy images. The first experiment was performed on a part of an image of the fresco “The Condemned in Hel” by Luca Signorelli and the second on a corrupted cDNA image, which are used for measuring the expression level of large number of genes. The restoration results are presented in Figure 15, and they confirm good denoising capabilities of the proposed IDCNN filter.

### 3.3. The Influence of Impulse Detection Imperfections

To confirm that the main source of error is the method of pixel replacement, we presented the Aim Diagram (AD), which separates the distribution of errors that were caused by improper classification of impulses. Using traditional metrics, we are not able to judge whether the main source of the error is incorrect impulse detection or corrupted pixels restoration.

**Figure 16:**
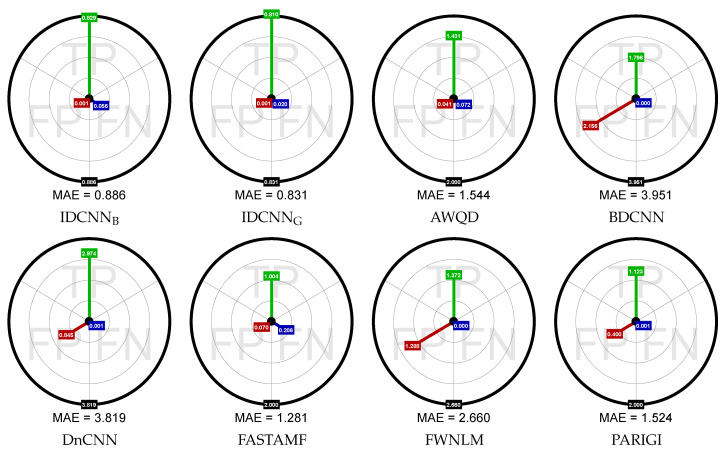
Diagrams that show what portion of the MAE error was caused by the improper decision of the used filter from classification perspective. These diagrams were obtained for the PEPPERS image (ρ = 0.4).

In the proposed diagrams, the radius in the circle denote the proportion of the MAE metric calculated independently for pixels that are TP, FP and FN, respectively. The error for TN pixels is equal to zero and therefore it is not presented in the plots. The AD calculated for the MAE metric is presented in Figure 16.

The networks IDCNN_B_ and IDCNN_G_, trained on BSD500 and GoogleV4 datasets respectively, almost perfectly detected the impulses, and the main contribution to the MAE error comes from insufficient quality of the restoration of the noisy pixels. For the proposed method, the total MAE for the analyzed PEPPERS image is equal to 0.886, while error related to incorrectly detected impulses equals 0.056. In this way, the main contribution to the total error is made by the replacement (interpolation) of the correctly detected impulses. It shows that in further research, the efficiency of the proposed filter could be significantly improved if we used a better noisy pixel substitution method.

### 3.4. Computational Complexity

**Figure 17:**
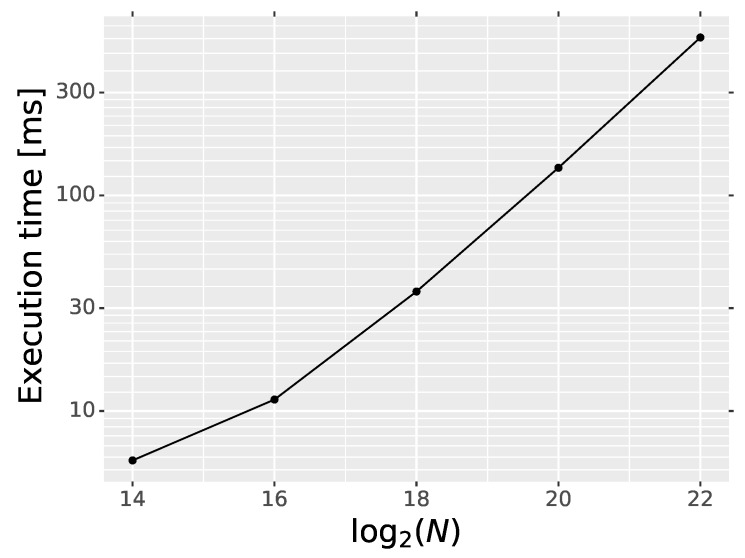
Execution time dependency of the IDCNN on the number of image pixels *N*.

All the experiments were performed in Tensorflow v1.8 [75] environment and Python client, running on a PC with Intel^®^ Core™ i7-3930K CPU 3.20 GHz and GeForce GTX 1080 Ti. In this setup, the training of the IDCNN detector with the default parameters (see Table 1) takes about six hours on GPU. Regarding the time for inference, we measured only the execution time of operations calculated on GPU, excluding the time required for data image reading and writing. The execution time is summarized in Table 8. As can be observed, execution time of the proposed IDCNN detector on GPU is similar to DnCNN, which is not surprising because both networks differ only slightly on the last layer. The execution time dependency of the proposed IDCNN on the number of image pixels is depicted in Figure 17. The time required for processing of an image is proportional to the number of pixels *N*.

## 4. Conclusions

In this work, we have introduced a switching filter that employs a deep neural network for impulsive noise removal in color images. The performed experiments reveal that the proposed filtering architecture, which operates using a modified version of DnCNN for impulsive pixels detection and adaptive mean filter for their restoration, outperforms or is comparable with the state-of-the-art filters in terms of PSNR, MAE and SSIM_c_ quality measures.

The proposed IDCNN filter performs well on images contaminated by artificial impulsive noise and also on those affected by real noise process, keeping the undistorted pixels unchanged, without introducing visible artifacts. Apart from the high filtering efficiency, the proposed method is quite fast, and processing of an image of the standard 512×512 size takes about 35 milliseconds on GPU, which enables applying our technique for real time applications. Moreover, the execution time is proportional to the number of pixels, which allows to efficiently process images also in higher resolutions. The unique feature of the introduced switching filter is that it does not require any adjusting parameters, and the same filtering framework can be applied regardless of the image contamination density. In this way, the proposed method can be applied in many denoising scenarios, as no user’s intervention is required. This is a crucial feature of our design, as the methods that deliver good results in terms of objective and also subjective evaluation, need to be tuned, which is mostly difficult and requires experience of the operator.

Future work will be continued in two main directions. The first goal will be the improvement of the efficiency of the noisy pixel replacement using other suitable CNNs. Thus, a combination of the proposed IDCNN for impulse detection and a CNN for noisy pixel restoration will significantly increase the overall performance of noisy image enhancement. The performed experiments indicate that the BDCNN output [72] could be used for the restoration of the detected noisy pixels. Another research direction will be focused on the elaboration of a single network able to combine both the detection and noisy pixel restoration in one processing stage.

## Figures and Tables

**Table 1:** Summary of the network parameters.

Parameter	Value/Method
Number of convolutional layers	17
Number of filters in convolutional layer	64
Size of convolutional window	3×3
Number of epochs	50
Learning rate	0.001
Learning rate decay	0.1
Epoch in which learning rate decay is used	30
Batch size	128
Weights initialization	Glorot uniform initializer [59]
Weights optimization	ADAM optimizer [60]
Patch size	41×41

**Table 2:** Repeatability of the training on BSD500 dataset in terms of weighted accuracy (wACC), False Positive Rate (FPR), False Negative Rate (FNR) and F_1_.

	Training Repetition										
	**Average wACC**						**Average F_1_**				
ρ	1	2	3	4	5		1	2	3	4	5
0.1	0.9985	0.9987	0.9987	0.9985	0.9987		0.9926	0.9935	0.9933	0.9923	0.9937
0.2	0.9984	0.9986	0.9986	0.9985	0.9985		0.9960	0.9965	0.9965	0.9962	0.9963
0.3	0.9980	0.9981	0.9982	0.9981	0.9980		0.9966	0.9969	0.9970	0.9968	0.9967
0.4	0.9970	0.9971	0.9972	0.9970	0.9970		0.9962	0.9963	0.9965	0.9963	0.9963
0.5	0.9945	0.9944	0.9944	0.9941	0.9944		0.9945	0.9944	0.9944	0.9940	0.9944
	Average FPR						Average FNR				
ρ	1	2	3	4	5		1	2	3	4	5
0.1	0.0014	0.0013	0.0013	0.0015	0.0012		0.0018	0.0016	0.0015	0.0015	0.0018
0.2	0.0014	0.0012	0.0012	0.0014	0.0013		0.0025	0.0023	0.0022	0.0023	0.0024
0.3	0.0014	0.0012	0.0012	0.0013	0.0013		0.0035	0.0034	0.0033	0.0034	0.0034
0.4	0.0017	0.0014	0.0014	0.0013	0.0015		0.0051	0.0052	0.0050	0.0054	0.0051
0.5	0.0020	0.0018	0.0016	0.0015	0.0018		0.0090	0.0093	0.0095	0.0103	0.0094

**Table 3:** Impact of the patch size used in the training procedure on the detection performance of the network in terms of wACC. The best values are presented in bold font.

Average wACC							
	Patch size p×p						
ρ	9	11	21	31	41	51	61
0.1	0.1005	0.1026	0.9987	0.9986	0.9987	0.9985	**0.9988**
0.2	0.2063	0.2159	0.9985	0.9985	0.9985	0.9985	**0.9986**
0.3	0.5258	0.5887	0.9980	0.9980	**0.9981**	0.9980	0.9980
0.4	0.6003	0.6107	0.9969	**0.9972**	0.9971	0.9970	0.9969
0.5	0.5000	0.5005	0.9938	**0.9950**	0.9946	0.9944	0.9942

**Table 4:** Influence of the dataset type used in the training and its size on the average wACC. The best values are presented in bold font.

Average wACC							
**BSD500**							
	**Dataset Size**						
ρ	10	50	100	200	300	400	500
0.1	0.9910	0.9969	0.9983	0.9982	0.9985	0.9985	**0.9987**
0.2	0.9889	0.9962	0.9979	0.9982	0.9983	0.9984	**0.9986**
0.3	0.9830	0.9946	0.9970	0.9976	0.9978	0.9980	**0.9982**
0.4	0.9701	0.9909	0.9952	0.9962	0.9967	0.9970	**0.9972**
0.5	0.9434	0.9807	0.9898	0.9924	0.9938	**0.9945**	0.9944
VOC2007							
0.1	0.9906	0.9985	0.9985	0.9992	0.9993	0.9991	**0.9992**
0.2	0.9872	0.9977	0.9985	0.9989	0.9990	**0.9990**	**0.9990**
0.3	0.9798	0.9962	0.9977	0.9982	0.9984	0.9985	**0.9986**
0.4	0.9647	0.9931	0.9958	0.9967	0.9972	0.9973	**0.9974**
0.5	0.9345	0.9849	0.9894	0.9922	0.9930	0.9926	**0.9939**
GoogleV4 dataset							
0.1	0.9907	0.9980	0.9983	0.9990	0.9986	0.9990	**0.9993**
0.2	0.9850	0.9979	0.9983	0.9988	0.9987	0.9990	**0.9991**
0.3	0.9766	0.9973	0.9979	0.9985	0.9985	0.9987	**0.9988**
0.4	0.9614	0.9954	0.9964	0.9975	0.9975	0.9980	**0.9981**
0.5	0.9332	0.9886	0.9900	0.9932	0.9937	0.9950	**0.9952**

**Table 5:** Dependence of the noise density used during training using BSD500 dataset on the network performance and its ability to detect impulses in test images degraded with varying noise density. The  best values are presented in bold font.

Average wACC				
ρ	**Random**	**0.1**	**0.3**	**0.5**
0.1	0.9991	**0.9993**	0.9987	0.9973
0.2	**0.9985**	**0.9985**	**0.9985**	0.9971
0.3	0.9976	0.9962	**0.9981**	0.9969
0.4	0.9965	0.9868	**0.9971**	0.9965
0.5	0.9948	0.9520	0.9947	**0.9956**

**Table 6:** Comparison of the denoising efficiency of the proposed network for impulsive noise removal with the state-of-the-art methods on selected representative images from the test dataset [36]. The result obtained with the most efficient filter are emboldened and 5 best results are highlighted with green color.

ρ	IDCNN_B_	IDCNN_G_	AWQD	BDCNN	DnCNN	FAPGF	FASTAMF	FFNRF	FRF	FWNLM	INRF	L0TV	PARIGI	PD	PGF	QBSF	TSQSVF
FRUITS																	
PSNR [dB]																	
0.1	39.78	**40.35**	38.39	34.74	35.18	37.61	38.30	36.97	36.03	33.10	33.90	27.76	34.66	33.62	37.03	32.98	36.68
0.2	36.54	**37.24**	35.47	34.81	36.47	34.69	35.47	33.20	33.21	31.60	32.03	27.18	33.14	32.52	32.52	29.83	33.45
0.3	34.22	**35.00**	32.65	34.16	33.75	32.19	32.70	28.44	30.80	30.46	30.28	26.06	31.00	30.57	27.85	27.83	31.03
0.4	32.37	**33.20**	30.55	32.83	31.37	29.89	29.86	23.95	27.62	29.25	28.43	25.30	29.25	28.18	23.52	26.35	28.85
0.5	29.71	30.08	27.68	**30.63**	27.87	26.53	25.40	19.88	22.27	27.60	25.26	24.67	26.81	25.13	19.67	24.16	26.01
MAE																	
0.1	0.45	**0.42**	0.49	2.74	1.27	0.55	0.50	0.51	0.55	2.87	0.84	1.35	1.51	2.89	0.53	0.80	0.55
0.2	0.88	**0.84**	0.98	2.89	1.66	1.07	0.96	1.08	1.07	3.25	1.41	2.01	1.93	3.21	1.21	1.70	1.11
0.3	1.36	**1.30**	1.62	3.16	2.31	1.73	1.52	2.12	1.72	3.70	2.14	2.91	2.51	3.95	2.40	2.77	1.83
0.4	1.90	**1.81**	2.37	3.55	3.25	2.61	2.30	4.16	2.72	4.22	3.14	3.81	3.10	5.33	4.69	3.96	2.70
0.5	2.70	**2.65**	3.60	4.22	5.03	4.20	4.07	8.26	5.53	4.91	4.99	4.77	3.93	8.02	9.21	5.94	4.22
SSIM_c_																	
0.1	0.985	**0.987**	0.980	0.947	0.972	0.979	0.983	0.978	0.978	0.892	0.973	0.916	0.935	0.912	0.974	0.934	0.971
0.2	0.970	**0.974**	0.962	0.936	0.953	0.956	0.966	0.940	0.958	0.877	0.947	0.881	0.918	0.895	0.927	0.867	0.940
0.3	0.950	**0.958**	0.938	0.916	0.920	0.921	0.941	0.842	0.934	0.859	0.909	0.825	0.896	0.853	0.824	0.811	0.906
0.4	0.925	**0.936**	0.905	0.889	0.861	0.861	0.893	0.649	0.877	0.838	0.843	0.768	0.872	0.774	0.640	0.751	0.858
0.5	0.880	**0.886**	0.845	0.853	0.744	0.746	0.772	0.420	0.660	0.808	0.721	0.718	0.842	0.659	0.433	0.671	0.779
BALLOONS																	
PSNR [dB]																	
0.1	40.09	**40.91**	37.44	29.92	36.65	36.94	38.18	37.09	36.26	33.86	34.52	32.93	36.15	31.23	37.27	32.87	36.59
0.2	36.83	**37.75**	34.94	33.46	34.52	34.56	35.54	33.35	33.16	32.79	32.84	31.07	34.74	30.41	32.59	29.85	33.55
0.3	34.17	**35.13**	32.40	33.89	32.22	32.24	32.94	28.37	30.54	31.62	31.02	29.38	32.24	29.17	27.53	27.86	31.02
0.4	31.90	**33.30**	30.14	32.78	30.03	29.90	30.04	23.52	27.29	30.72	28.75	27.79	30.13	27.17	22.90	26.09	28.54
0.5	28.56	29.85	27.34	**30.97**	26.78	26.25	25.11	19.20	21.89	29.41	25.14	26.80	28.31	24.71	18.85	23.95	25.55
MAE																	
0.1	0.31	**0.29**	0.38	3.35	1.18	0.45	0.37	0.35	0.41	1.83	0.67	0.77	0.81	2.46	0.38	0.76	0.42
0.2	0.61	**0.58**	0.73	2.84	1.66	0.83	0.70	0.77	0.82	2.08	1.05	1.32	1.09	2.83	0.88	1.42	0.83
0.3	0.97	**0.91**	1.20	3.10	2.31	1.37	1.11	1.65	1.36	2.41	1.62	1.97	1.53	3.57	1.98	2.21	1.38
0.4	1.40	**1.26**	1.80	3.41	3.30	2.14	1.71	3.71	2.23	2.76	2.46	2.72	1.99	4.94	4.43	3.27	2.16
0.5	2.26	**1.99**	2.90	4.09	5.39	3.77	3.49	8.29	4.88	3.30	4.34	3.54	2.58	7.48	9.69	5.02	3.62
SSIM_c_																	
0.1	0.993	**0.994**	0.990	0.899	0.978	0.983	0.989	0.987	0.985	0.949	0.982	0.929	0.976	0.942	0.980	0.933	0.980
0.2	0.985	**0.986**	0.980	0.916	0.960	0.963	0.977	0.955	0.969	0.940	0.964	0.884	0.969	0.920	0.939	0.875	0.959
0.3	0.973	**0.977**	0.964	0.895	0.926	0.929	0.960	0.851	0.948	0.929	0.932	0.835	0.955	0.876	0.823	0.826	0.931
0.4	0.954	**0.966**	0.939	0.881	0.852	0.863	0.920	0.635	0.900	0.915	0.867	0.783	0.941	0.791	0.612	0.775	0.886
0.5	0.902	**0.923**	0.887	0.857	0.690	0.724	0.783	0.378	0.690	0.888	0.725	0.730	0.920	0.673	0.375	0.705	0.802
PEPPERS																	
PSNR [dB]																	
0.1	47.75	**47.88**	44.06	28.68	40.50	44.52	45.88	43.33	44.09	38.06	40.83	37.90	39.72	35.77	41.21	36.40	41.94
0.2	43.85	**44.41**	39.81	32.26	36.92	40.54	41.34	35.52	39.99	36.50	37.86	34.71	38.43	33.94	33.44	33.37	37.63
0.3	40.67	**41.64**	36.56	33.90	33.99	35.93	36.50	28.11	36.12	35.12	34.39	32.58	36.68	30.64	26.48	30.94	34.07
0.4	37.54	**39.16**	33.19	33.52	30.51	31.98	31.75	22.28	29.99	33.92	30.60	30.41	34.71	27.82	21.09	28.42	30.20
0.5	32.38	**34.02**	28.35	31.89	26.51	26.79	24.67	17.59	22.17	31.86	24.64	27.46	31.62	26.22	16.91	25.04	25.32
MAE																	
0.1	0.17	**0.17**	0.23	4.23	1.16	0.21	0.18	0.23	0.22	1.74	0.36	0.46	0.51	2.15	0.27	0.40	0.25
0.2	0.36	**0.35**	0.53	3.57	1.75	0.47	0.40	0.62	0.49	1.99	0.72	0.94	0.79	2.63	0.77	0.92	0.58
0.3	0.59	**0.57**	0.94	3.72	2.53	0.92	0.72	1.63	0.87	2.29	1.27	1.50	1.13	3.76	2.19	1.65	1.07
0.4	0.89	**0.83**	1.54	3.95	3.82	1.66	1.27	4.30	1.62	2.66	2.14	2.17	1.52	5.30	5.86	2.79	1.92
0.5	1.52	**1.34**	2.87	4.53	6.25	3.37	3.36	10.54	4.45	3.23	4.41	3.20	2.13	7.16	13.58	4.94	3.94
SSIM_c_																	
0.1	0.997	**0.997**	0.995	0.847	0.952	0.992	0.995	0.993	0.993	0.955	0.991	0.955	0.983	0.939	0.987	0.971	0.990
0.2	0.993	**0.994**	0.987	0.874	0.905	0.979	0.989	0.960	0.986	0.947	0.977	0.917	0.977	0.914	0.938	0.942	0.977
0.3	0.988	**0.990**	0.974	0.855	0.850	0.946	0.973	0.835	0.973	0.936	0.948	0.876	0.968	0.870	0.774	0.907	0.952
0.4	0.977	**0.984**	0.947	0.838	0.762	0.878	0.934	0.583	0.925	0.923	0.884	0.834	0.958	0.821	0.498	0.849	0.897
0.5	0.932	**0.954**	0.867	0.812	0.615	0.733	0.773	0.317	0.694	0.891	0.714	0.783	0.941	0.748	0.275	0.745	0.765

**Table 7:** Comparison of the denoising efficiency of the proposed network for impulsive noise removal with the state-of-the-art methods on the test dataset [36]. The result obtained with the most efficient filter are emboldened and 5 best results are highlighted with green color.

ρ	IDCNN_B_	IDCNN_G_	AWQD	BDCNN	DnCNN	FAPGF	FASTAMF	FFNRF	FRF	FWNLM	INRF	L0TV	PARIGI	PD	PGF	QBSF	TSQSVF
**Average PSNR [dB]**																	
0.1	40.12	**40.45**	38.10	31.91	38.92	36.78	37.97	36.20	37.22	32.39	34.82	31.27	34.33	34.32	36.68	31.89	36.28
0.2	37.02	**37.36**	34.93	33.22	36.18	34.11	35.06	32.38	33.88	31.22	32.60	29.34	32.75	32.33	31.88	28.85	33.10
0.3	34.77	**35.26**	32.50	32.66	33.64	31.80	32.53	27.77	31.08	30.22	30.62	27.91	31.15	29.91	27.20	26.96	30.68
0.4	32.68	**33.38**	30.14	31.35	30.86	29.22	29.33	23.20	27.28	29.23	28.16	26.65	29.52	27.01	22.84	25.32	28.25
0.5	29.92	**30.51**	27.29	29.70	27.52	25.92	24.77	19.20	21.71	28.03	24.73	25.48	27.78	23.93	19.09	23.42	25.45
Average MAE																	
0.1	0.46	**0.45**	0.57	3.74	1.18	0.72	0.60	0.72	0.59	2.76	1.01	1.38	1.62	2.35	0.63	0.93	0.61
0.2	0.92	**0.90**	1.14	3.48	1.71	1.31	1.12	1.34	1.21	3.30	1.65	2.24	2.18	2.99	1.40	1.96	1.24
0.3	1.42	**1.39**	1.84	3.91	2.41	2.02	1.72	2.44	2.01	3.90	2.45	3.16	2.82	4.13	2.75	3.18	2.03
0.4	2.01	**1.94**	2.77	4.51	3.49	3.04	2.60	4.78	3.30	4.58	3.59	4.20	3.58	6.10	5.50	4.72	3.13
0.5	2.86	**2.75**	4.24	5.38	5.45	4.80	4.59	9.48	6.56	5.39	5.66	5.38	4.53	9.46	10.75	7.08	4.94
Average SSIM_c_																	
0.1	0.989	**0.989**	0.984	0.895	0.980	0.977	0.983	0.975	0.981	0.915	0.974	0.922	0.949	0.946	0.976	0.927	0.974
0.2	0.978	**0.979**	0.968	0.916	0.962	0.955	0.967	0.940	0.961	0.897	0.949	0.875	0.931	0.913	0.929	0.860	0.947
0.3	0.963	**0.966**	0.945	0.902	0.932	0.920	0.944	0.841	0.935	0.876	0.913	0.825	0.908	0.849	0.815	0.798	0.912
0.4	0.944	**0.949**	0.910	0.876	0.872	0.856	0.898	0.645	0.875	0.850	0.848	0.773	0.881	0.746	0.624	0.732	0.859
0.5	0.904	**0.911**	0.845	0.838	0.748	0.736	0.771	0.414	0.666	0.815	0.718	0.717	0.845	0.615	0.420	0.649	0.771

**Table 8:** Comparison of average execution time (in miliseconds) of the proposed IDCNN detector and DnCNN method on GPU.

Image Size	$\log_{2}N$	DnCNN	IDCNN
128×128	14	5.6	5.9
256×256	16	11.3	11.3
512×512	18	36.3	35.8
1024×1024	20	134.3	134.2
2048×2048	22	546.3	539.5
